# Effects of integrated chronic care models on hypertension outcomes and spending: a multi-town clustered randomized trial in China

**DOI:** 10.1186/s12889-017-4141-y

**Published:** 2017-03-11

**Authors:** Yuting Zhang, Wenxi Tang, Yan Zhang, Lulu Liu, Liang Zhang

**Affiliations:** 1 Department of Health Policy and Management, Graduate School of Public Health, University of Pittsburgh, 130 De Soto St, Pittsburgh, PA 15261 USA; 2 Department of Pharmacoeconomics, School of International Pharmaceutical Business, China Pharmaceutical University, 639 Longmian St, Jiangning District, Nanjing, 211198 China; 3 School of Medicine and Health Management, Tongji Medical College, Huazhong University of Science and Technology, 13 Hangkong Road, Wuhan, Hubei 430030 China; 4 Department of Economics, University of Pittsburgh, 230 S. Bouquet St, Pittsburgh, PA 15260 USA; 5 Research Center for Rural Health Service, Key Research Institute of Humanities & Social Sciences of Hubei Province, Wuhan, Hubei 430030 China

**Keywords:** Integrated chronic care model, Clustered randomized trial, Hypertension, China

## Abstract

**Background:**

Hypertension affects one billion people globally and is one of the leading risk factors for cardiovascular and renal diseases. However, hypertension management remains poor, especially in rural China.

**Methods:**

A clustered randomized controlled trial was conducted in six towns in China’s Qianjiang county between 7/2012 and 6/2014, including 5462 hypertension patients above 35 years old. Six towns were randomly assigned to three groups: Group 1 had the integrated care model including a multidisciplinary team and continuous care coordination, Group 2 had both the integrated care model and provider-level financial incentives, and the control group had the usual care. Primary outcomes were systolic blood pressure and health-related quality of life measured by SF36; secondary outcomes included hypertension-related hospitalization rate and inpatient spending. Blood pressure was measured sixteen times bimonthly between 12/1/2011 and 6/30/2014, and quality of life was measured on 7/1/2012 and 6/30/2014. Inpatient data between 7/1/2010 and 8/31/2014 were used. This trial is registered at the World Health Organization’s International Clinical Trials Registry, number ChiCTR-OOR-14005563.

**Results:**

We found that the integrated care model effectively lowered blood pressure by 1.93 mmHg (95% CI 0.063–3.8), improved self-assessed health-related quality of life, and reduced the rate of hypertension-related hospitalization by 0.17 percentage points (95% CI 0.094–0.24). We also found that the provider-level financial contract further lowered blood pressure by 1.76 mmHg (95% CI 0.73–2.79) and reduced rates of hospitalization and inpatient spending, but it also reduced patients’ self-assessed health-related quality of life.

**Conclusions:**

Integrated care and financial incentives are effective in lowering blood pressure and reducing hospitalization rate, but financial contracts may hurt patient quality of life.

This trial was registered at the Chinese Clinical Trial Registry (ChiCTR-OOR-14005563) on November 23, 2014. It was a retrospective registration.

## Background

As the burden of disease has shifted from acute illness to chronic diseases worldwide, patients’ needs require more continuous care coordination and integration across multiple professional providers. However, in the current healthcare delivery system, patients often experience fragmented medical care, and the fragmentation of care has become one of the major challenges many countries face [1, 2]. To reform the fragmented system, new integrated care models, with or without new funding models, are being tried out around the world, including American Accountable Care Organizations, Australian Primary Health Networks, British Integrated Care Pioneers, and the Germany’s Gesundes Kinzigtal model [1, 3, 4].

Healthcare is particularly fragmented in rural China, where the healthcare system includes three tiers: village, town, and county [5]. Patients go to village clinics for simple outpatient care and preventive care, and go to town hospitals or county hospitals for inpatient care or outpatient conditions village clinics cannot handle. Communications among multiple professional providers are limited and there are virtually no document sharing and interactions among providers across the three tiers. Thus, an integrated care model shows particular promise for improving care in China. However, no large-scale interventions have been conducted to test alternative chronic-care models that integrate care across providers and across tiers in rural China.

Some new integrated care models being tested out in western countries include a new funding component in which providers can share savings if spending falls below a pre-specified financial benchmark. There is some early evidence showing that these types of financial incentives are associated with potential savings in total healthcare costs [3, 4, 6]. However, in general, fewer studies have evaluated effects of provider-level financial incentives on chronic care management, especially through randomized control trials [7, 8].

In this study, we conducted a multi-town clustered randomized trial on hypertension management in rural China. Six towns were randomly assigned to three groups: Group 1, with the integrated care model including a multidisciplinary team and continuous care coordination; Group 2, with both the integrated care model and provider-level financial incentives; and a control group, with usual care.

Patients in our study sample had been diagnosed with hypertension for at least 6 months and had three or more measures of blood pressure greater than 140/90 mmHg in the Chinese national official medical record before our interventions began. Hypertension is prevalent, affecting nearly one billion people worldwide and over 442 million people in China [9, 10]. It is one of the leading risk factors for cardiovascular and renal diseases and is estimated to cause 12.8% of total deaths worldwide and 20% of deaths in China [9, 10]. In China, the proportion of patients with hypertension unaware of their conditions was over 30% and the proportion of known hypertensive patients who received treatment was only 56.1% in 2012 [11, 12]. The rate of uncontrolled blood pressure (defined as blood pressure greater than 140/90 mmHg) among Chinese with hypertension is much higher than in the West, estimated to be 78.3% in 2008 and 63.4% in 2012 [11, 13]. In addition, inequality in hypertension management is prevalent in China: poor or rural residents were much less likely to receive treatment, compared to those living in urban or affluent areas [11]. For all these reasons, there are potentially large health gains from validating better hypertension treatment models in rural China.

Previous studies have found that inadequate control of hypertension is often related to gaps in continuity of health care [10], poor adherence to medications [14], and unhealthy lifestyle [10], and integrated care can improve hypertension management [15]. For example, Glynn and colleagues reviewed 72 clinical trials designed to improve hypertension management in primary care settings and concluded that patients benefited from an organized system of regular patient follow-up and a multidisciplinary team to improve coordination of care [15]. An integrated healthcare delivery system in northern California has demonstrated an effective program to manage hypertension, often considered as best practice [16]. This program includes a comprehensive hypertension registry, standardization of blood pressure measurements, an evidence-based treatment algorithm, and a multidisciplinary team including medical assistants, nurses, and pharmacists [16]. Several recent review articles note that multidisciplinary team-based care for hypertension management consistently results in improved blood pressure [17–20], and may be cost-effective [21].

Our work therefore builds upon evidence from previous studies, but two contributions of our study are especially distinctive: First, to our knowledge, this is the first large-scale randomization to study integrated care and provider financial incentives in China. We therefore provided some first-hand evidence of how the integrated care model and the financial contract can be incorporated in less developed areas where inadequate medical resources, fragmented care, and poor quality of care are prevalent. Second, previous interventions were often randomized at clinic or provider level, while as we relied on a distinctive randomization at the town level. This design choice is important because it enables us to evaluate the integration across village-town-county tiers in rural China, for the first time.

## Methods

### Study setting and usual care

**Fig. 1:**
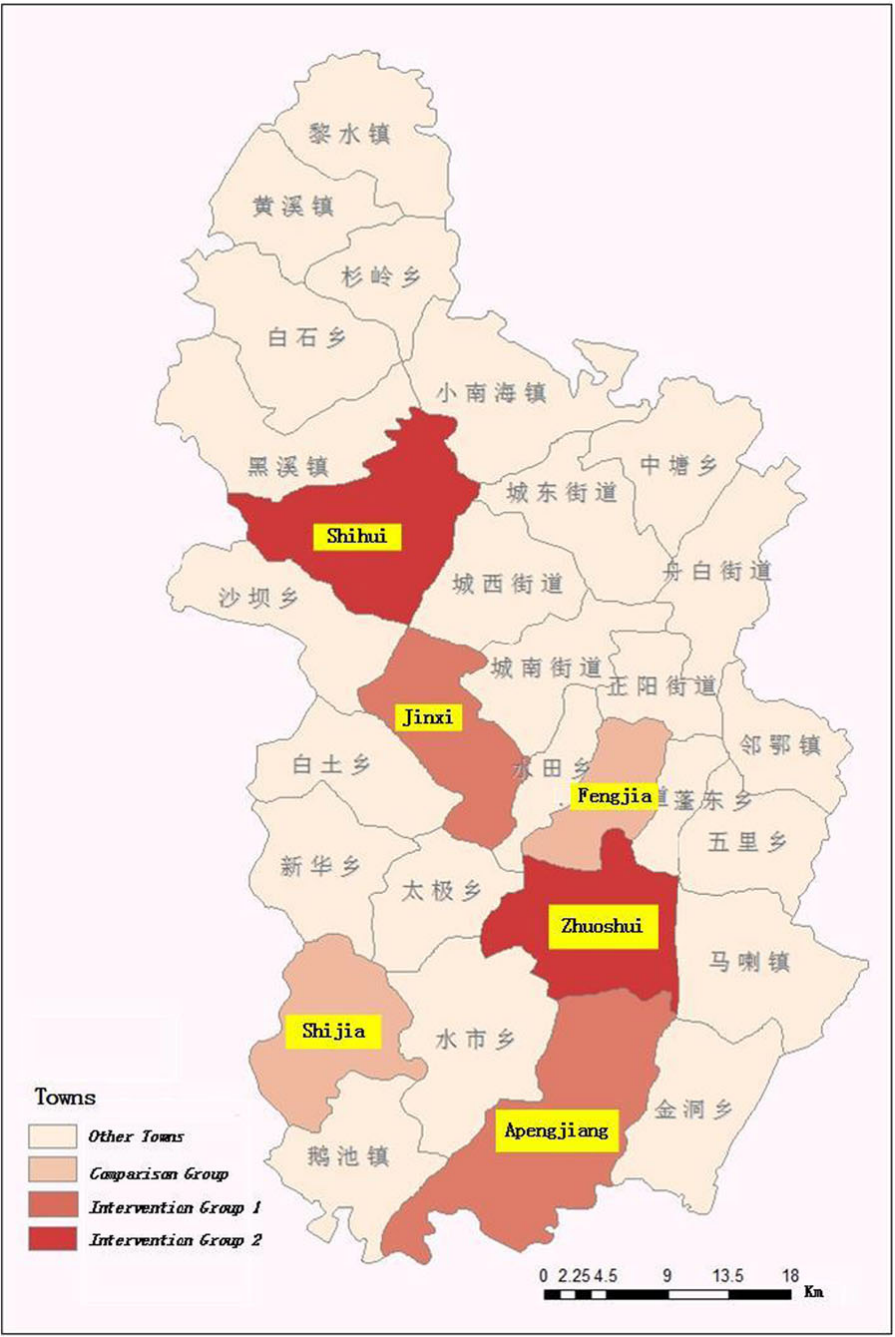
Geographic Locations of Six Towns. Notes: Intervention Group 1 consisted of Jinxi and Apengjiang; intervention Group 2 consisted of Zhuoshui and Shihui; and the control group consisted of Shijia and Fengjia. Sources: authors created it

Our intervention sites were in Qianjiang county, a typical rural area that is 280 km to the south east of Chongqing, one of the largest cities and one of four municipalities in China. Qianjiang has a population of 550,000 people, with an average annual income per capita of under USD480 for the past 5 years [22]. Qianjiang has 30 communities, 24 of which are rural towns and the remaining six are urban districts (Fig. 1). The average town population is approximately 18,000, and each town consists of around 10 villages.

Rural residents obtain their health insurance through the New Rural Cooperative Medical Scheme, which provides health insurance for 627 million Chinese rural residents, accounting for 46% of the total Chinese population as of 2014 [23]. Rural residents seek care from a village-town-county three-tier healthcare delivery system. A typical village has one clinician with limited medical care training, who provides patient education, simple outpatient care and preventive care to all villagers. The village clinic, sometimes in the clinician’s home, has a small supply of medications but often lacks rudimental medications that are included in the national essential medicine list. There are no hospitals in the villages. Each town has one hospital and rural residents go to town hospitals for inpatient care and outpatient care that the village clinic is not equipped to handle.

If town hospitals cannot treat the patient’s illness, the patient goes to county or city hospitals. Under the current system, in most areas, patients can also go to county or city hospitals directly without going through town hospitals, but the inpatient reimbursement rate is much lower for services provided in county or city hospitals compared to town hospitals. For example, in 2012 in Qianjiang, the inpatient policy reimbursement rate was 70% for services provided in town hospitals and 55% in county hospitals. The actual effective reimbursement rate in county hospitals was even lower, about 44% in 2012, because many services provided in county hospitals were not eligible for reimbursement. Regardless of where rural residents in Qianjiang seek their inpatient care (town, county or city), the reimbursable portion of the medical care is paid with social health insurance pooled at Qianjiang county level. The gross costs of an inpatient stay in a county hospital can easily double the cost in town hospitals for the same procedure, and the difference in costs between city and county hospitals is even larger. Residents are incentivized to seek care in lower level hospitals because of their lower gross costs and higher reimbursement rate.

### Interventions

We tested two new care components: the integrated care model started on Aug 1, 2012, and the financial incentive contract started on Jun 1, 2013, (because it took some time to negotiate contracts), in comparison with usual care. Both interventions ended on Jun 30, 2014. We conducted a clustered randomized controlled trial with two intervention groups (Group 1 and Group 2) and a control group (usual care). Group 1 only received the integrated care model, while Group 2 received both the integrated care model and the financial incentives. Thus, we use Group 1 to test the improvements in integrated care that could be made within the current funding model, and Group 2 to test the additional effects of the financial incentives.

### Integrated care model

Our integrated care model for chronic conditions has two important features: one is to combine treatment and prevention care with a multidisciplinary team; the other is to provide continuous care coordination across the village-town-county three-tier delivery system.

#### Multidisciplinary team

Each patient in our sample potentially has a team of 27 providers, consisting of 23 clinicians and four non-clinical staff. The team includes the patient’s village clinician (each village only has one clinician), two physicians (one outpatient and one inpatient) and four non-clinicians (three chronic disease coordinators/educators, and one case manager) at the town hospital (each town hospital has three to four physicians and four non-clinician staff to treat patients with hypertension), and 20 clinicians at the county hospitals. Qianjiang has two public county-level hospitals; from each hospital, we enrolled ten doctors: two outpatient physicians and three inpatient physicians from each of the two departments: neurology and cardiology. Each country hospital has about 16 to 20 doctors to treat hypertension patients.

#### Continuous care coordination

We designed three mechanisms through which continuous care could be effectively provided to patients. First, patient medical records, lab results, and other health related documents were shared among all participating providers. Second, each month, county doctors gave lectures to township clinicians on how to diagnose and treat patients with hypertension and conducted case reviews on selected patients. Town doctors then provided monthly lectures and discussed cases with village doctors. The group learning was recorded by the case manager and assessed later by the grant project manager. Third, we categorized four different types of clinical pathways depending on the severity of hypertension (Appendix). In each pathway, we specified clear guidelines on definitions of severity, on measurements town physicians should provide before referring to county clinicians, and on what county clinicians could and could not do based on the reports from village and town providers.

### Financial contracts

A contract was signed with each of two towns in Group 2 on Jun 1, 2013. The contract clearly stated that if at the end of the performance year, the total actual inpatient spending for all patients with hypertension in Group 2 towns was above the benchmark amount, the providers who participated in the trial would be paid according to the regular reimbursement schemes; however, if the total actual inpatient spending was below the predicted benchmark amount, they would obtain a bonus at 60% of total savings.^1^ The provider team is responsible for the total actual inpatient spending for all patients in the Group 2 towns, regardless whether the patients seek care at town, county or city hospitals. Thus, healthcare providers cannot reduce cost through patient selection. Because inpatient spending is much higher in upper-level hospitals than lower-level hospitals, providers have incentives to encourage patients to use lower-level hospitals.

### Randomization at the town level

The head of the Department of Health in Qianjiang identified six out of 24 rural towns as feasible for this type of interventions, according to population size, socioeconomic development, and capacity levels of medical facilities. We divided the six towns equally into two clusters: one cluster of three towns with a smaller population, lower socioeconomic development levels, poorer hospital quality, and further distance away from the county center; and the other cluster with contrasting characteristics. In each cluster, we randomly assigned three towns to Groups 1, 2 and the control group. If a town was selected, all villages in the town were automatically selected for the study. Figure 1 shows the geographic locations of 6 towns and Table 1 compares towns’ basic characteristics that were surveyed in Jul 2012.

**Table 1:** Comparison of Characteristics Used to Randomly Assign Six Towns To Three Groups, July 2012

	Intervention Group 1		Intervention Group 2		Control Group	
	Apengjiang	Jinxi	Zhoushui	Shihui	Fengjia	Shijia
Number of residents	28,000	15,600	27,055	22,448	27,464	14,126
Annual average income per capita (CNY)	6452	5417	6487	5452	6900	5031
Distance to county (minutes)	50	90	30	60	25	100
Medical facility revenue (CNY10K)	265	169	279	198	311	159

Notes: These basic characteristics were surveyed in July 2012 and used to randomly assign towns to the three groups. We first divided the six towns equally into two clusters: one cluster of three towns with a smaller population, lower socioeconomic development levels, poorer hospital quality, and further distance away from the county center (Jinxi, Shihui, and Shijia); and the other cluster with contrasting characteristics. In each cluster, we randomly assigned three towns to Groups 1, 2 and the control group, respectively

### Randomization at the patient level

We obtained the entire list of patients residing in the six towns who had been officially registered as a chronic patient with hypertension in the national chronic disease database between Jan 1, 2008, and Jan 1, 2012. Not everyone with hypertension was registered during this period. Having a national official medical record means that these patients were aged 35 and over, had a history of hypertension for at least 6 months, had blood pressure recorded at least four times a year, and had at least three measures of blood pressure greater than 140/90 mmHg. Our list includes 5,789 patients. We obtained all of their inpatient claim data between Jan 1, 2010, and Dec 31, 2014.

To collect more person-level blood pressure, health related quality of life, and life style information through in-person in-house survey, we randomly selected 300 patients from each town, because we did not have resources to interview all 5789 patients. We stratified a random sample on the basis of three variables: gender (female and male), age (35–60, 61+), and risk level of hypertension (high, medium, and low). Using these three variables, we stratified 12 mutually exclusive subgroups per town and selected patients by subgroup. Before random sampling, we excluded patients who: 1) had a stable blood pressure history (consistently under 120/80 mmHg) for longer than 1 year because they did not need to take medications; 2) were estimated for a life expectancy less than 2 years due to old age, vegetative state or severe complications such as cerebral infarction or pancreatic cancer; 3) were considered to be difficult to follow up because they were away for over 6 months a year due to migration for work, education or caring for family; and 4) were unable to complete surveys due to intellectual incompetence, mental damage or inability to communicate.

Among 1800 randomly-sampled patients, 1641 patients agreed to participate and conducted the baseline interview (91.2%), but only 1408 had valid complete baseline survey data (85.8%), 1,245 (75.9%) of which also completed the end-point interview and had valid data. The response rate was relatively high because village clinicians who know patients well accompanied the interviewers to patient homes and subjects were provided some household staples (toothpastes, soap, etc.) as gifts if they agreed to participate in the study.

### Data sources and outcomes

We used three main data sources: inpatient claims data for all 5,789 patients, patient-level survey data and blood pressure measures from the national chronic condition management registry database for 1,245 randomly selected patients. Inpatient data did not include death information, so we obtained death information from the Qianjiang Health Bureau but we could not get death records before 2012.

For patient-level survey data, we had two points: one measured at the baseline on Jul 1, 2012, and the other measured at the end-point on Jun 30, 2014. Systolic blood pressure was measured bimonthly between Dec 1, 2011, and Jun 30, 2014, for four times in the baseline period and 12 times post interventions.

In order to test each intervention and because inpatient data is highly volatile, we consolidated inpatient data into ten 5-month periods between Jul 1, 2010, and Aug 31, 2014: five periods in the baseline before intervention 1, two periods between interventions 1 and 2, and three periods after intervention 2. For those who died during our intervention period, we followed them up until they died. The unit of observations is person-period. For each person-period, we calculated three outcomes to measure hypertension related hospitalization: rates of hospitalization (1 = any hypertension related inpatient stay; 0 = otherwise), likelihood of using a county/city hospital, and total gross inpatient spending. We define hypertension related hospitalization as hospitalization related to hypertension as well as other conditions for which hypertension is a risk factor, including cardiovascular diseases and stroke.

Health related quality of life was measured with in-house surveys using the Medical Outcome Study Short-Form 36-Item Health Survey (SF36 Scale) that was developed by the RAND Corporation Health Insurance Experiment [24]. In reporting results, we primarily focused on three summary scores: SF36 physical health, psychological health, and total scores. Some subjects had missing values on these variables, so we only had 1,082 subjects with both baseline and end-point measurements.

### Covariates

Covariates included age, gender, family structure (living alone, with spouse, with children only, with spouse and children, or with others), education (no education, elementary and middle school, high school and above), average annual income, average annual medical expenditure, salt and fat control, and self-assessed competence in treatment adherence. These were collected through in-person surveys at both baseline and the end-point.

### Statistical analyses

**Fig. 2:**
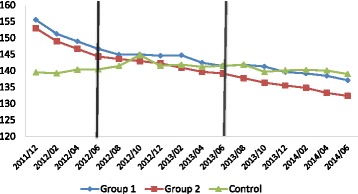
Trend in Unadjusted Systolic Blood Pressure by Group

We plotted the trend in blood pressure by group for all 16 measures we have between Dec 2011 and Jun 2014 (Fig. 2). Baseline comparisons on other measures were undertaken by Chi-squared test for categorical variables and one-way analysis of variance for continuous variables. Because towns in our intervention groups faced considerable amount of administrative pressures to lower systolic blood pressure to below 140 mmHg, they had initiated some strategies to lower blood pressure before our interventions started. These strategies included providing free medications, imposing more rigid supervision of village doctors, and designating someone in the town hospital to manage chronic conditions. At the start of our interventions, however, these strategies were stopped as a result of our negotiations with participating towns’ administration team. Figure 2 shows that the control group had a different baseline trend from the intervention groups in the 8 months leading to our interventions, but blood pressure levels across three groups became comparable at the beginning of our interventions.

We used difference-in-differences models to test the effects of both interventions simultaneously. Specifically, we included the post intervention 1 indicator (≥Aug 1, 2012), the indicator for being exposed to intervention 1 (individuals in group 1 and group 2), their interaction term, the post intervention 2 indicator (≥Jun 1, 2013), the indicator for being exposed to intervention 2 (individuals in group 2), and their interaction term. Our variables of interest are the two interaction terms, which represent the marginal effects of the two interventions.

Because the baseline trends in blood pressure before our interventions were different between intervention groups and the control group, to avoid overestimating the intervention effects on blood pressure, we included linear bi-month time trends in the model. In addition, we also conducted sensitive analyses to only use data points between Jun 2012 and Jun 2014, and report this as our primary results as lower bound. The model also included individual fixed effects to control for unobserved characteristics within individuals and time fixed effects.

For health-related quality of life, we estimated a similar difference-in-differences model that included the post intervention indicator, the indicator for being exposed to intervention 1, the indicator for being exposed to intervention 2, and the two interaction terms between post intervention indicator and intervention exposure indicators. The model also controlled for individual fixed effects and time-variant covariates mentioned above.

For three outcomes on hypertension related hospitalizations, we conducted three separate models; in each model, we controlled for time and town fixed effects, and used individual random effects instead of individual fixed effects, because many individuals had zero inpatient visits. First, we used logistic regression models to estimate the intervention effects on rates of hospitalization. Second, to estimate the utilization rate for town or county/city hospitals, we adopted a two-part model which consisted of two logistic regressions: we first estimated the rate of having any hospital stays and then estimated the rate of using county/city hospitals conditioning on having any hospital stays. Third, we evaluated the effect on inpatient spending using a two-part model: first, we estimated the use of hospitalization with a logistic regression, and second, we estimated effects on inpatient spending conditional on having an inpatient stay using a generalized linear model with gamma distribution and logarithm link. We then estimated the marginal effects and the standard errors of the interaction terms using bootstrap [25].

The project protocol was approved by the Tongji Medical College Academic Ethics Committee of Huazhong University of Science and Technology in China (IORG No: IORG0003571). This trial is registered at the Chinese Clinical Trials Registry (ChiCTR-OOR-14005563).

## Results

Table 2 compares individual characteristics at the July 2012 baseline across three groups and shows that most characteristics are comparable. Individuals in Group 1 were slightly more educated and had higher self-assessed competence in treatment adherence compared to those in the other two groups. Individuals in Group 1 had slightly poorer health measured by SF36 scores compared to the control group, but health in Group 1 and Group 2 was similar. There was no statistically significant difference in overall health between Group 2 and the other two groups.

**Table 2:** Comparison of Characteristics by Group at the Baseline, July 2012

Variables	Group 1	Group 2	Control	Group 1-Control	Group 2-Control	Group 1-Group 2
Age	64.5	66.5	65.5	−0.9	1.1	**−2.0**
Female, %	51.9	54.2	55.9	−4.0	−1.7	−2.2
Family structures, %						
Living alone	14.4	16.0	14.6	−0.2	1.3	−1.5
Living with spouse only	34.3	32.8	36.9	−2.7	−4.2	1.5
Living with kids only	13.4	17.6	17.3	−3.9	0.4	−4.2
Living with both spouse and kids	35.8	32.8	28.8	**7.0**	4.0	3.1
Other family structure	2.1	0.8	2.4	−0.3	**−1.6**	1.2
Education, %						
No education	32.4	37.2	39.6	**−7.2**	−2.4	−4.8
Attend elementary school	45.8	48.5	44.4	1.4	4.1	−2.7
Attend high school or above	21.9	14.3	16.1	**5.8**	−1.8	**7.6**
Average annual income, ¥	5540	6797	5405	135	**1392**	−1257
Personal annual medical expenditure, ¥	1854	1689	1891	−37	−202	165
Salt control	3.5	3.4	3.3	0.2	**0.2**	0.0
Fat control	3.7	3.5	3.6	0.1	0.0	0.2
Self-assessed competence to treatment adherence	75.4	71.8	70.7	**4.7**	1.1	**3.6**
SF36 Physical health	45.4	47.3	50.5	**−5.1**	−3.1	−1.9
SF36 Psychological health	48.3	50.8	52.8	**−4.5**	−2.0	−2.5
SF36 Total Score	47.8	50.1	52.5	**−4.7**	−2.4	−2.3

Notes: Bold denotes significant at *p*-value < 0.05. The values of salt control and fat control are 1 = never, 2 = occasional, 3 = sometimes, 4 = often, 5 = always; self-assessed competence to treatment adherence is between 0 and 100, with a larger number indicating higher competence; and the values of SF scores are between 0 and 100, with a larger number indicating better health

Table 3 shows the estimated effects of interventions on the level of systolic blood pressure from two difference-in-differences models, one with data points between Dec 2011 and Jun 2014 and the other between Jun 2012 and Jun 2014 as our primary results to be more conservative.

**Table 3:** Effects of Interventions on Systolic Blood Pressure from the Difference-in-differences Model

	(1)	(2)
Integrated Care Model	−4.77***	−1.93**
	(0.85)	(0.95)
Financial Contract Model	−1.59***	−1.76***
	(0.56)	(0.53)
No. of observations	19,965	16,221
Time Trend	Yes	Yes
Data starting time	Dec/2011	Jun/2012

Notes: This table shows the marginal effects of the two interventions using a difference-in-differences model. Individual fixed effect and linear time trends are included to adjust for the non-parallel pre-intervention trend. Robust standard errors are in parentheses; *** denotes *p* < 0.01, ** denotes *p* < 0.05

Relative to the control group, the integrated care model was associated with a 1.93 mmHg (95% CI 0.063–3.8; *p* < 0.001) reduction in systolic blood pressure, or 1.3% of the pre-intervention level at 149.3 mmHg. Relative to the integrated care model, financial incentives were associated with an additional 1.76 mmHg (95% CI 0.73–2.79; *p* < 0.001) reduction in systolic blood pressure, or 1.2% of the pre-intervention level.

**Fig. 3:**
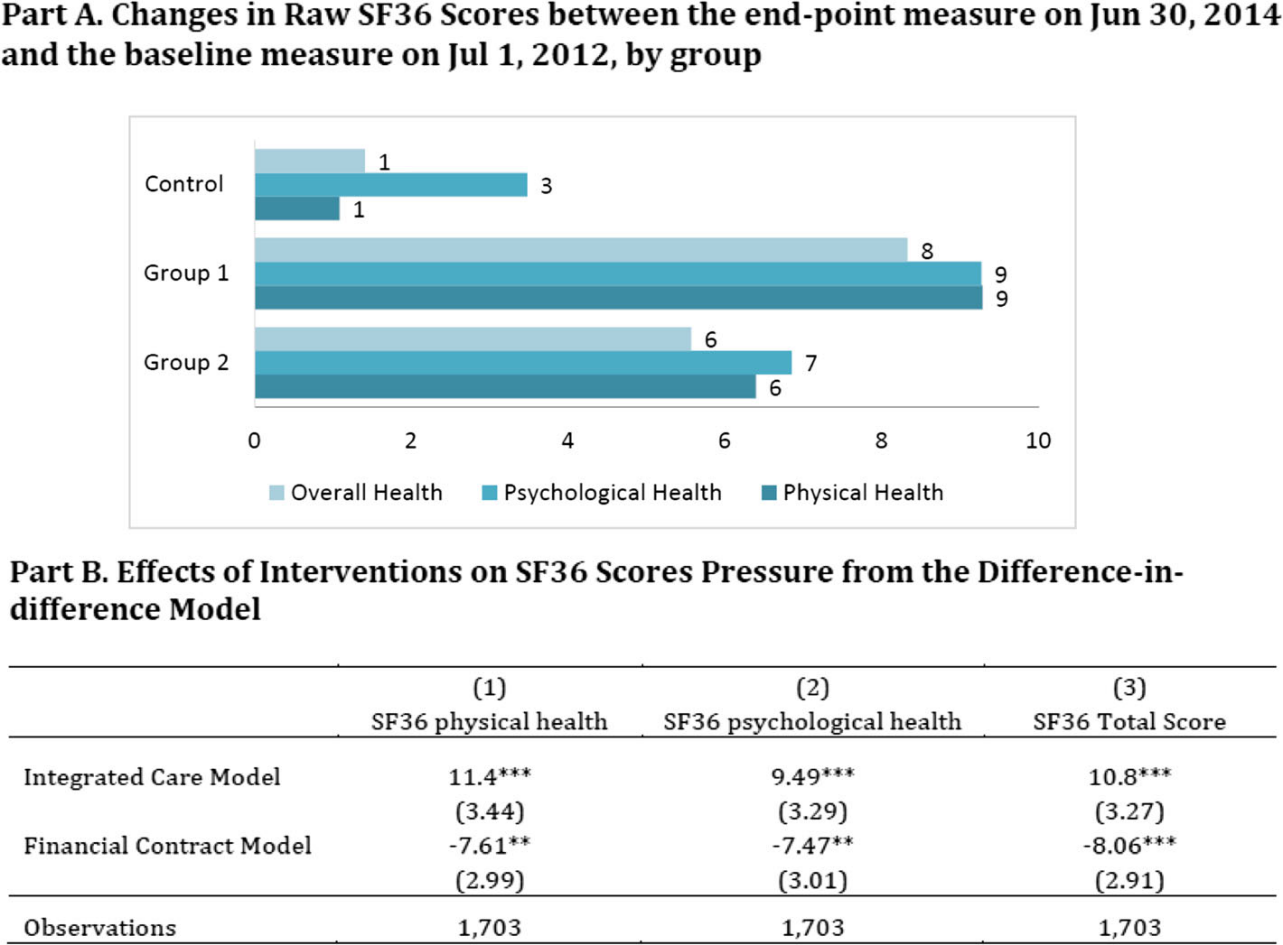
Effects of Interventions on Quality of Life Measured by Three SF36 Scores. Notes: This table shows the marginal effects of the two interventions using a difference-in-differences model. All SF36 scores range between 0 and 100, with larger numbers indicating better life quality. Individual fixed effects are included. Robust standard errors are in parentheses; *** denotes *p* <0.01, ** denotes *p* <0.05

Figure 3 shows the estimated results on health-related quality of life. Integrated care improved health outcomes measured by SF36 scores: it increased physical health by 11.4 points, psychological health by 9.5 points, and overall health by 10.8 points. However, the intervention of provider-level financial incentives was associated with a worse self-assessed health-related quality of life: overall health is lowered by 8.06 points.

Table 4 shows the effects of interventions on three outcomes on hypertension-related hospitalization: hospitalization rate, likelihood of using a county hospital, and total spending. As mentioned above, inpatient data were aggregated per person every 5 months. In the baseline period from Jul 1, 2010, to Jul 31, 2012, the average rate of hypertension-related hospitalization per person per 5 months was 1.9% in Group 1 and Group 2, and 1.8% in the control group.

**Table 4:** Marginal Effects of Interventions on Overall Hospitalization Rate, Rate of Using Upper Level Hospitals, and Inpatient Spending for Hypertension Related Hospitalization, Bootstrapping Results from Two-step Difference-in-differences Models

	(1)	(2)
	Integrated Care Model	Financial Incentives
Hospitalization rate	−0.00167***	−0.000955***
	(0.000373)	(0.000338)
Likelihood of using an upper level hospital	0.000494*	−0.000188
	(0.000270)	(0.000333)
Total inpatient spending	9.398***	−5.208***
	(1.654)	(1.354)
No. of observations	57,890	57,890

Notes: Robust standard errors are in parentheses; *** denotes *p* < 0.01, and * denotes *p* < 0.1. All models included fixed effects for town and time period, as well as individual random effect

We observed a consistent pattern that both interventions resulted in lower rates of hypertension-related hospitalization. Specifically, the integrated care model reduced the rate of hospitalization by 0.17 percentage points (95% CI 0.094–0.24), or 8.9% from the baseline 1.9 percentage points per person every 5 months in Group 1 and 2 (Table 4 Column 1). The financial incentives further reduced the rate of hospitalization by 0.096 percentage points (95% CI 0.029–0.16), or 5.1% from the Group 2 baseline level (Table 4 Column 2).

The likelihood of using a county level hospital increased slightly due to integrated care but did not change with additional financial incentives. Integrated care was associated with a slight increase in total inpatient spending, by $9.40 (95% CI 6.16–12.64) per person every 5 months, or 13.3% from the baseline 70.89. This can be partially explained by more use of county level hospital instead of town hospitals because gross costs in the former more than doubled gross costs in the latter. Financial incentives were associated with lower total inpatient spending, by $5.21 (95% CI 2.55–7.86), or 7.9% from the baseline 65.68 in Group 2.

## Discussion

We found that the integrated care model effectively lowered blood pressure, improved self-assessed health-related quality of life, and reduced the rates of hypertension-related hospitalization, but we did not find that the integrated care model reduced total inpatient spending, partially because integrated care increased the use of county-level hospitals instead of town hospitals. We also found that the provider-level financial contract further lowered blood pressure and reduced rates of hospitalization and inpatient spending, but it also reduced patients’ self-assessed health-related quality of life.

These results are encouraging, given over half of hypertensive patients currently have uncontrolled blood pressure globally [11, 26, 27]. Our interventions lowered systolic blood pressure, to an extent on the lower spectrum of findings in previous studies [15, 17, 18, 20, 28, 29]. Our observed effects are small for two main reasons: first, our interventions target on healthcare system delivery changes, which may take a long time to realize effects. Second, subjects in our study had much higher baseline blood pressure than those in previous studies and therefore are more difficult to treat, However, for subjects in our study, even a small reduction in blood pressure could result in meaningful effects on lowering hospitalizations and cardiovascular risk. Indeed, we observed that interventions reduced the rate of cardiovascular related hospitalization. A recent meta-analysis reviewed nineteen clinical trials and confirmed the effects of lowering blood pressure on reducing major cardiovascular events, myocardial infarction, stroke, albuminuria, and retinopathy progression [30]. More importantly, our subjects are mainly farmers, and physical health has large and direct effects on their income, and therefore the improved hypertension control has more economic impact which is not measured here.

It is interesting to observe that provider-level financial incentives are associated with reductions in patient self-assessed quality of life. When provider payments are aligned with total inpatient spending, providers may discourage patients from using hospital services, especially more expensive services in higher-level hospitals. If patients do not trust their doctors, which is very common in China, patients may think they were not provided the best medical treatment and thus feel worse about their own health or simply report worse health than they perceive.

Our study is not without limitations. First, the financial contract in Group 2 was not fully executed. By design, the potential bonus to providers was tied with outcome-based performance measures and savings in total inpatient spending in the town. However, in reality, the assessment on performance was weak and not fully monitored. Second, our interventions were conducted in one county in China, and therefore results may not be generalizable to other areas. However, Qianjiang county is highly representative of China’s rural areas, in terms of its population size, mobility, economic development state, and prevalence rates of chronic diseases. In addition, we obtained our patient list from the national chronic disease registry for hypertension, and patients officially registered in the registry were sicker than general patients ever diagnosed with hypertension.

## Conclusions

Because our study groups were randomly assigned, our findings are guarded against some selection biases that are common in other recent evaluations of integrated care models that were conducted through observational data [3, 4]. Taken together, our findings have important clinical and policy implications on how to better manage chronic conditions. First, the integrated care model substantially lowered blood pressure and improved patients’ health. Our findings support changing from the current fragmented care model to the more integrated, multidisciplinary-team-based care model, even in the current funding model. This result is consistent with other integrated care models that are being tried out in other countries. Second, continuous care coordination across provider disciplines and village-town-county tiers was important; so was sharing medical records among providers and developing continuous quality improvement processes. Third, combining integrated care with a new funding model further reduced blood pressure and inpatient spending, but also reduced patients’ quality of life. This suggests that financial incentives should not be too quickly introduced in rural China without further evaluations.

Because the relatively low costs (USD25K) and potential savings to conduct the interventions, it is feasible to roll out the integrated care model to other rural areas in China more broadly. Most rural areas in China have the financial ability to support such a new delivery model. The key to the success is the central government’s willingness to try and the local administration team’s leadership to implement it.

In sum, the integrated care model and the financial contract are effective in lowering blood pressure and reducing rates of hypertension-related hospitalization. Integrated care is also associated with better health-related quality of life. However, the long-term savings in healthcare costs from these models remain to be seen.

## Appendix

### Four types of clinical pathways in the integrated care model

Type A for which patients first sought care in their village clinics, was a minor condition that could be treated by village clinicians. If the condition did not improve, patients were referred to town hospitals. Type B was an emergency case, defined as instant blood pressure ≥ 180/120 mmHg, and if blood pressure was not reduced by 25% within an hour, the patient was admitted to the emergency room. Type C was a complex and serious condition with complications, defined by the following criteria: 1) instant blood pressure ≥ 180/120 mmHg; 2) severely damaged target organs; and/or 3) other cardiovascular, cerebrovascular and circulatory system complications. Once village or town doctors identified a Type B or C patient, they had to contact their corresponding county hospital clinicians and transfer the patient to the relevant county hospital department. During the inpatient stay, county doctors needed to monitor the patient status and also followed up after inpatient discharge. Type D patients were newly identified patients with hypertension and had a visit directly in a county hospital without going through the clinical pathway. Once a type D patient was identified, the patient was enrolled in our integrated care pathway system, and the county provider contacted the local village clinician to start a new medical record for the patient.

## Footnotes

1 During the intervention period, the operations of the social health insurance in Qianjiang were reorganized from county level to city level and the Qianjiang county health bureau no longer had authority to change the reimbursement policy. As a result, although the contract stated a bonus to be 60% of the savings, the actual bonus at the end of the project was set by the head of the Qianjiang administrative office as CNY70K (USD10,654) per town in Group 2, and it was actually paid through the research grant to be shared among 23 clinicians and four non-clinical staff. However, during the intervention period, providers were not aware of this change, so they were still subject to the incentives in the contracts.

## References

[1] Hall J (2015). Australian health care — The challenge of reform in a fragmented system. N Engl J Med.

[2] O’Connor SJ (2014). Fragmentation is a prominent feature of the American healthcare landscape. J Healthc Manag.

[3] Busse R, Stahl J (2014). Integrated care experiences and outcomes in Germany, the Netherlands, and England. Health Aff (Millwood).

[4] Nyweide DJ, Lee W, Cuerdon TT (2015). Asociation of pioneer accountable care organizations vs traditional medicare fee for service with spending, utilization, and patient experience. JAMA.

[5] Yip W, Hsiao W (2014). Harnessing the privatisation of China’s fragmented health-care delivery. Lancet.

[6] Dorr DA, McConnell KJ, Williams MP, Gray KA, Wagner J, Fagnan LJ, Malcolm E (2015). Study protocol: transforming outcomes for patients through medical home evaluation and redesign: a cluster randomized controlled trial to test high value elements for patient-centered medical homes versus quality improvement. Implement Sci.

[7] de Bruin SR, Baan CA, Struijs JN (2011). Pay-for-performance in disease management: a systematic review of the literature. BMC Health Serv Res.

[8] Petersen LA, Simpson K, Pietz K, Urech TH, Hysong SJ, Profit J, Conrad DA, Dudley RA, Woodard LD (2013). Effects of individual physician-level and practice-level financial incentives on hypertension care: a randomized trial. JAMA.

[9] Feng XL, Pang M, Beard J (2014). Health system strengthening and hypertension awareness, treatment and control: data from the China Health and Retirement Longitudinal Study. Bull World Health Organ.

[10] Centers for Disease Control and Prevention (2011). Vital signs: prevalence, treatment, and control of hypertension--United States, 1999–2002 and 2005–2008. MMWR Morb Mortal Wkly Rep.

[11] Hou Z, Meng Q, Zhang Y. Hypertension prevalence, awareness, treatment, and control following China’s healthcare reform. Am J Hypertens. 2015. 10.1093/ajh/hpv125 PMC4886484 26232034

[12] Wang Y, Peng X, Nie X, Chen L, Weldon R, Zhang W, Xiao D, Cai J. Burden of hypertension in China over the past decades: systematic analysis of prevalence, treatment and control of hypertension. Eur J Prev Cardiol. 2016;23(8):792–800. 10.1177/2047487315617105 26603746

[13] Lewington S, Lacey B, Clarke R, et al. The Burden of Hypertension and Associated Risk for Cardiovascular Mortality in China. JAMA internal medicine 2016;176(4):524–32. 10.1001/jamainternmed.2016.0190 26975032

[14] Margolis KL, Asche SE, Bergdall AR, Dehmer SP, Maciosek MV, Nyboer RA, O’Connor PJ, Pawloski PA, Sperl-Hillen JM, Trower NK (2015). A successful multifaceted trial to improve hypertension control in primary care: why did it work?. J Gen Intern Med.

[15] Glynn LG, Murphy AW, Smith SM, Schroeder K, Fahey T (2010). Interventions used to improve control of blood pressure in patients with hypertension. Cochrane Database Syst Rev.

[16] Jaffe MG, Lee GA, Young JD, Sidney S, Go AS (2013). Improved blood pressure control associated with a large-scale hypertension program. JAMA.

[17] Houle SK, Chatterley T, Tsuyuki RT (2014). Multidisciplinary approaches to the management of high blood pressure. Curr Opin Cardiol.

[18] Proia KK, Thota AB, Njie GJ, Finnie RK, Hopkins DP, Mukhtar Q, Pronk NP, Zeigler D, Kottke TE, Rask KJ (2014). Team-based care and improved blood pressure control: a community guide systematic review. Am J Prev Med.

[19] Fortuna RJ, Nagel AK, Rose E, McCann R, Teeters JC, Quigley DD, Bisognano JD, Legette-Sobers S, Liu C, Rocco TA (2015). Effectiveness of a multidisciplinary intervention to improve hypertension control in an urban underserved practice. J Am Soc Hypertens.

[20] Walsh JM, McDonald KM, Shojania KG, Sundaram V, Nayak S, Lewis R, Owens DK, Goldstein MK (2006). Quality improvement strategies for hypertension management: a systematic review. Med Care.

[21] Jacob V, Chattopadhyay SK, Thota AB, Proia KK, Njie G, Hopkins DP, Finnie RK, Pronk NP, Kottke TE (2015). Economics of team-based care in controlling blood pressure: a community guide systematic review. Am J Prev Med.

[22] Chinese National Economic and Social Development Statistics. Qianjiang District National Economic and Social Development Statistics Bulletin 2001–2012.2013.

[23] Rural population (% of total population) - China [http://data.worldbank.org/country/china]. Accessed 9 Feb 2017.

[24] Medical Outcomes Study: 36-Item Short Form Survey Scoring Instructions [http://www.rand.org/health/surveys_tools/mos/mos_core_36item_scoring.html]. Accessed 9 Feb 2017.

[25] Ai C, Norton E (2003). Interaction terms in Logit and Probit models. Econ Lett.

[26] Egan BM, Zhao Y, Axon RN (2010). US trends in prevalence, awareness, treatment, and control of hypertension, 1988–2008. JAMA.

[27] Guessous I, Bochud M, Theler JM, Gaspoz JM, Pechere-Bertschi A (2012). 1999–2009 Trends in prevalence, unawareness, treatment and control of hypertension in Geneva, Switzerland. PLoS One.

[28] Clark C, Smith L, Cloutier L, Glynn L, Clark O, Taylor R, Campbell J (2015). Lb01.01: allied health professional-Led interventions for improving control of blood pressure in patients with hypertension: a Cochrane systematic review and meta-analysis. J Hypertens.

[29] Shaw RJ, McDuffie JR, Hendrix CC, Edie A, Lindsey-Davis L, Nagi A, Kosinski AS, Williams JW (2014). Effects of nurse-managed protocols in the outpatient management of adults with chronic conditions: a systematic review and meta-analysis. Ann Intern Med.

[30] Xie X, Atkins E, Lv J, Bennett A, Neal B, Ninomiya T, Woodward M, MacMahon S, Turnbull F, Hillis GS (2016). Effects of intensive blood pressure lowering on cardiovascular and renal outcomes: updated systematic review and meta-analysis. Lancet.

